# A new instrument to measure healthy workplace qualities: the People in the Office Scale

**DOI:** 10.3389/fpsyg.2023.1241555

**Published:** 2023-11-02

**Authors:** Milada V. Pavlova, Sofia I. Reznichenko, Sofya K. Nartova-Bochaver

**Affiliations:** School of Psychology, HSE University, Moscow, Russia

**Keywords:** healthy workplace, psychological well-being, assessment, employee, evidence-based design, questionnaire, office

## Abstract

This research is aimed at developing a new instrument to assess the healthy workplace qualities based on the environmental theory of stress, and ideas of salutogenic and biophilic design. A total of 319 respondents participated in the study (19–72 years; M_age_ = 40.86, SD_age_ = 12.70; 69% women). Additionally, nine judges were invited to evaluate the items of the scale for content validity. Using a mixed inductive/deductive method, which included literature analysis and in-depth interviews, an initial pool of 56 items was collected. From the initial pool of 56 items, the more relevant ones were selected. This list named the *People in the Office Scale (POS)* was subjected to a full psychometric examination. Results of the Exploratory and Confirmatory Factor Analyses show that *POS* has satisfactory structural and content validity, reliability, and measurement invariance across sex and age. In its final form, *POS* includes 27 items and five subscales: *Ergonomics*; *Internal Communications*; *External Infrastructure*; *Freedom of Action*; and *Workplace as a Life Narrative*. Convergent validity measured by correlating *POS* scores with the variables of restorative environment, workplace attachment, and organizational cynicism was satisfactory. Divergent validity measured by correlating with mental health, was also satisfactory. This new instrument can be recommended for use in both practice and research to provide evidence-based design guidance.

## 1. Introduction

People spend a lot of time at work; in the office, they solve many different functional tasks, not only production ones. Thanks to the development of positive psychology, the social attitude towards specialists has also become more humane. The pragmatic perception of an individual as a producer of public goods only has been replaced by a humanistic understanding of employees as the agents of their professional lives. Employees study and socialize, order food delivery, communicate with children and even pets, sometimes sleep, and get massages or beauty treatments in the office. Hence, the work environment is closely related to the personal needs of employees. That is why the task of organizational psychology is to develop a healthy work environment promoting not only high labor efficiency but also specialists’ well-being and optimal functioning.

A healthy work environment (an ideal universal workplace) allows a person associate their personal narrative with this place (Colenberg et al., 2021; Bergefurt et al., 2022; Haapakangas et al., 2022). This understanding is in line with a definition of a healthy workplace given by World Health Organization and Burton (2010). A healthy workplace helps the staff, firstly, protecting and promoting their health, safety and well-being, and secondly, the sustainability of the organization as integrity. In a healthy work environment, priorities are health and safety concerns in the physical and psychosocial work environment, personal health resources, and participating in the community to improve the health of workers, their families and relatives. Below, we use the word “office” as a synonym for “workplace,” because, although some authors consider it outdated (Vischer, 2008), it is more familiar to a wide range of specialists. In addition, the construction “in office” has one more meaning shade – to be at work, regardless of whether a person works remotely or even at home, which determined the title of our tool.

The concept of the workplace has evolved beyond the traditional office to include various environments like hospitals, universities and etc. Moreover, the boundaries between home and work spaces are often blurred in many professions, with employees even preferring the workplace over their homes (Hochschild, 2003; Damaske et al., 2014; Banga and Mahajan, 2021; Vandelannoitte, 2021). Sometimes, especially due to COVID-19 lockdown, the home has taken on office-like characteristics, while office spaces now accommodate various activities beyond production tasks. Thus, the classic ergonomic view of the workplace is already outdated today.

Following the environmental theory of stress (Edwards et al., 1998; Armitage and Nassor Amar, 2021; De Cooman and Vleugels, 2022), the workplace should fit the employee’s personality. Recently, the explanation of the origin of stress and coping with it through environmental influences has become both generalized and effective (Altomonte et al., 2020; Bluyssen, 2022).

Taking into account the high importance of the physical environment for employees’ well-being and resilience of the organization, the lack of research instruments to measure the workplace characteristics is surprising. Existing tools largely focus on the organizational and physical activities (Chau et al., 2012; Duncan et al., 2013; Jancey et al., 2014; Alonso Nuez et al., 2022) or social relations and corporate culture (Scrima, 2015; Razak et al., 2016; Pacheco and Coello-Montecel, 2020). Perceived Remote Workplace Environment Quality Indicators, a tool recently developed by Mura et al. (2023) measures both tangible workspace characteristics and subjective comfort perception but it is limited to remote work settings only. The Workplace Attachment Scale measures employees’ emotional bond to the office only, without representations of the workplace characteristics. All of the above demonstrates the urgent need to develop a new research tool based on the understanding of the employee as a healthy, resilient agent of their life and self-realization. Salutogenic and biophilic designs are two ways to reach this aim. Salutogenic design (Dilani, 2008; Golembiewski, 2016, 2022; Forooraghi et al., 2022) prioritizes employees’ physical and mental health by developing supportive work environments. Design solutions in this context aim to enhance a person’s “sense of coherence” contributing to health and well-being, while reducing stress-inducing environmental demands (Antonosky, 1996; Roskams and Haynes, 2019; Bergefurt et al., 2022). Salutogenic design implies personal control over lighting, temperature, sound, and space, plus the provision of both formal and informal meeting areas, quiet rooms and features encouraging physical activity. Biophilic design (Kellert and Calabrese, 2015; Browning and Cooper, 2016; Al-Dmour et al., 2021) emphasizes the inherent human connection with nature (biophilia) and incorporates natural elements into built environments (Browning et al., 2014). Designers incorporate natural materials, adequate ventilation, natural lighting, and views, alongside associations with natural phenomena for a multi-sensory experience (Kellert and Calabrese, 2015; Clements-Croome et al., 2019). This approach fosters physical and emotional well-being by creating an emotive space that increases workers’ attachment to their workspaces. To build a healthy workspace, evaluating work environment based on environmental stress theory, salutogenic and biophilic designs is vital. The aim of our research is to develop a new standardized instrument meeting the requirements of these approaches. We titled this tool *the People in the Office Scale (POS).*

## 2. Method

### 2.1. Participants

In total, 319 respondents participated in the study (19–72 years; M_age_ = 40.9, Me_age_ = 39, SD_age_ = 12.7; 220 women), mostly from Russia (87%), followed by Israel (6%), and New Zealand (2%), with all being Russian speakers. The participants were employed in government organizations (16%), small businesses (26%), medium companies (34%), and large businesses (24%), and held positions ranging from ordinary employees (24%) to top managers (9%). Their work experience varied from 6 months to 47 years. Average time spent at work was 39.3 h per week (SD = 13.4). We collected data during the pandemic but our respondents were asked about working in the office. We have not specially asked them about remote work; in a private conversation, some of them said that at the moment the work was temporarily remote. Inclusion criteria for the sample were being over 18 years old, working in an office setting, and having at least 6 months of work experience in the company.

In addition, 9 judges (architects and psychologists selected among people with high experience in the field of architecture/office design or positive/organizational psychology) evaluated the items of the initial pool, being informed about the aim of developing a new method. They participated in the stage of the items selection only. Data was collected by means of 1 ka.si service between 2021 and 2022.

### 2.2. Item Pool development and analytical strategy

An initial item pool was developed by means of a mixed-methods approach – deductive/inductive strategy (Kelly et al., 2013; Gönülateş, 2019). An extensive literature review was undertaken to identify workplace affordances related to employees’ somatic and mental health maintaining, restoration, inspiration, emotional regulation, and communication (Heerwagen et al., 1995; Kaplan, 1995; Burton et al., 2005; Dilani, 2008; Allen et al., 2017; Timm et al., 2018; Clements-Croome et al., 2019; Golembiewski, 2022; Rasheed and Rotimi, 2022). Various aspects of the workplace, such as location, infrastructure, and design, were considered as well, which resulted in the list of the statements describing a healthy workplace. This list was combined with the items extracted from the in-depth interviews discovered employees’ work style, personal development, social interactions, and relaxation preferences (Pavlova and Nartova-Bochaver, 2020). An initial set included 56 items (see Supplementary Table 1). For use outside of Russia, these items were translated into English by bi-lingual psychologists, based on the ISPOR recommendations (Wild et al., 2005). Based on the expert evaluation outcomes (nine judges), the content validity of 56 items was assessed using the Content Validity Ratio (CVR), according to the formula proposed by Lawshe (Polit and Beck, 2006). The items were considered essential if the experts assigned a score of 4 or 5.

We used Exploratory Graph Analysis (EGA), conducted within the glasso estimation method, and the Walktrap algorithm to identify the optimal number of subscales in the questionnaire and to estimate which items belong to each dimension. EGA was conducted using data from 127 (40% of the total sample) respondents.

Confirmatory factor analysis (CFA) with the robust maximum likelihood (MLR) estimator was performed to analyze the factor structure of *POS*. The set of commonly used goodness-of-fit indicators was used to interpret the results of CFA: CFI, TLI, RMSEA, PCLOSE, and SRMR (Hu and Bentler, 1999). A Multi-Group Confirmatory Factor Analysis (MG CFA) was carried out to assess the measurement invariance of the scale’s factor structure across sex and age. Evaluation of the invariance was conducted by the assessment of changes in the fit index: ΔCFI and ΔTLI less than 0.01, ΔRMSEA less than 0.015, and ΔSRMR less than 0.03 (Chen, 2007).

The internal reliability of the tool was estimated with the McDonald’s omega (ω): ω threshold values 0.70 are considered as acceptable (Hair et al., 2010).

CFA, MG CFA, and internal reliability testing were conducted on the CFA sample (*N* = 182; 60% of the total sample). Descriptive statistics and external construct validity was examined on the entire sample. The internal construct validity of the scales was assessed using the CFA sample data by examining the average variance extracted (AVE) and comparing the square root of the AVE to inter-construct correlations (Cohen, 1988).

We used the packages psych 2.2.9 (Revelle, 2022), lavaan 0.6–12 (Rosseel, 2022), semTools 0.5–6 (Jorgensen et al., 2022), EGAnet 1.2.3 (Golino and Christensen, 2022), and ccpsyc 0.2.7 (Fischer and Karl, 2019). The calculations were performed both in MS Excel and R Software v. 4.2.2 (R Core Team, 2022).

### 2.3. Measurement instruments

Three measures were chosen to examine convergent validity of a new tool.

1. *The Organizational Cynicism Scale* (*OCS*; Brandes et al., 1999; Russian version: Pavlova et al., 2022) evaluates employees’ negative attitudes towards the organization with 13 items on three subscales measured by a five-point Likert scale. In the current study, Cronbach’s alphas for the total *OCS* score, and *Cognitive*, *Emotional*, and *Behavioral Dimensions* of organizational cynicism were 0.91, 0.92. 0.90, and 0.81, respectively.
2. *The Perceived Restorativeness Scale* (*PRS*; Hartig et al., 1997; Russian version: Pavlova et al., 2022) measures the office environment’s restorative qualities with 15 items on *Being Away*, *Fascination*, and *Compatibility* subscales; an 11-point Likert scale was used. In the current study, Cronbach’s alphas for the total PRS score, and *Being Away, Fascination*, and *Compatibility* subscales were 0.94. 0.83, 0.91, and 0.94, respectively.
3. *The Office Attachment Scale (OAS)* is a modification of the *Place Attachment Scale* (Williams and Vaske, 2003; Pavlova et al., 2022) for the workspace; it assesses emotional attachment to the workplace through a unidimensional scale of 3 items measured by a five-point Likert scale. In the current study, Cronbach’s alpha was 0.89.

For divergent validity, the *Warwick-Edinburgh Mental Well-Being Scale (WEMWBS)* was used. It evaluates an individual’s self-reported mental wellbeing during the last 2 weeks (Tennant et al., 2007; Russian version: Robinson et al., 2013). It is a uni-dimensional scale consisting of 14 items regarding positive mental health. Respondents used a five-point Likert scale to answer. In the current study, Cronbach’s alpha was 0.90.

## 3. Results

### 3.1. Item analysis

The results of checking the content validity of the items, carried out using the method of expert assessments, showed that the CVR critical value (the lower level of agreement that exceeds chance levels at *p* ≤ 0.05) for nine expert assessments was 0.78. Thirteen out of 56 items had CVRs less than 0.78. The lowest value was obtained for the item “The office space allows people to grow their favorite plants if you wish” (CVR = −0.11); all other “irrelevant” items had CVRs ranging from 0.33 to 0.56. Despite the low content validity, we kept all of the items for further analysis because some of them consider the workplace as a source of professional identity (Bauer, 2020) whereas some others describe workspace settings of employees performing shift work practices.

Initial sample data (*N* = 319) had no missing values. 10 (3.1%) outliers have been removed based on Mahanobilis Test results (*p* < 0.001). Most items showed a slight bias towards higher scores. A few items had a bias toward low scores and a potential floor effect – 13, 29, 33, 34, 37, and 38. Both multivariate and univariate normality were not met (*p* < 0.001). Distributions were mostly negatively asymmetric (73.21%) and platykurtic (78.57%). However, in terms of the absolute values, the range of skewness and Pearson’s kurtosis were acceptable to prove normal univariate distribution (Gravetter and Wallnau, 2014) and did not exceed ±2, with the exception of item 31, which greatly exceeded the allowable range: Sk = −2.008, Ku = 5.143 (see Supplementary Table 2). Under the Classical Test Theory framework, we also analyzed item difficulty and discrimination indices, inter-item, and item-total correlations. The majority of items (78.57%) had acceptable difficulty (0.3–0.7). Items 29, 33, 34, and 37 were classified as “difficult” (<0.3), while items 2, 4, 9, 15, 19, 30, 31, and 32 as “easy.” Discrimination for most items was acceptable (>0.2; mean item discrimination coefficient: 0.40); only 8 items (1, 2, 3, 4, 5, 15, 31) had marginal discrimination indices (0.20–0.29; Kartik and Neeraj, 2013). The average inter-item correlation was 0.33, suggesting that the items are reasonably homogenous and contain sufficiently unique variance. The item-total correlations for all item scores ranged from 0.321 to 0.797.

### 3.2. Scale evaluation

**Figure 1:**
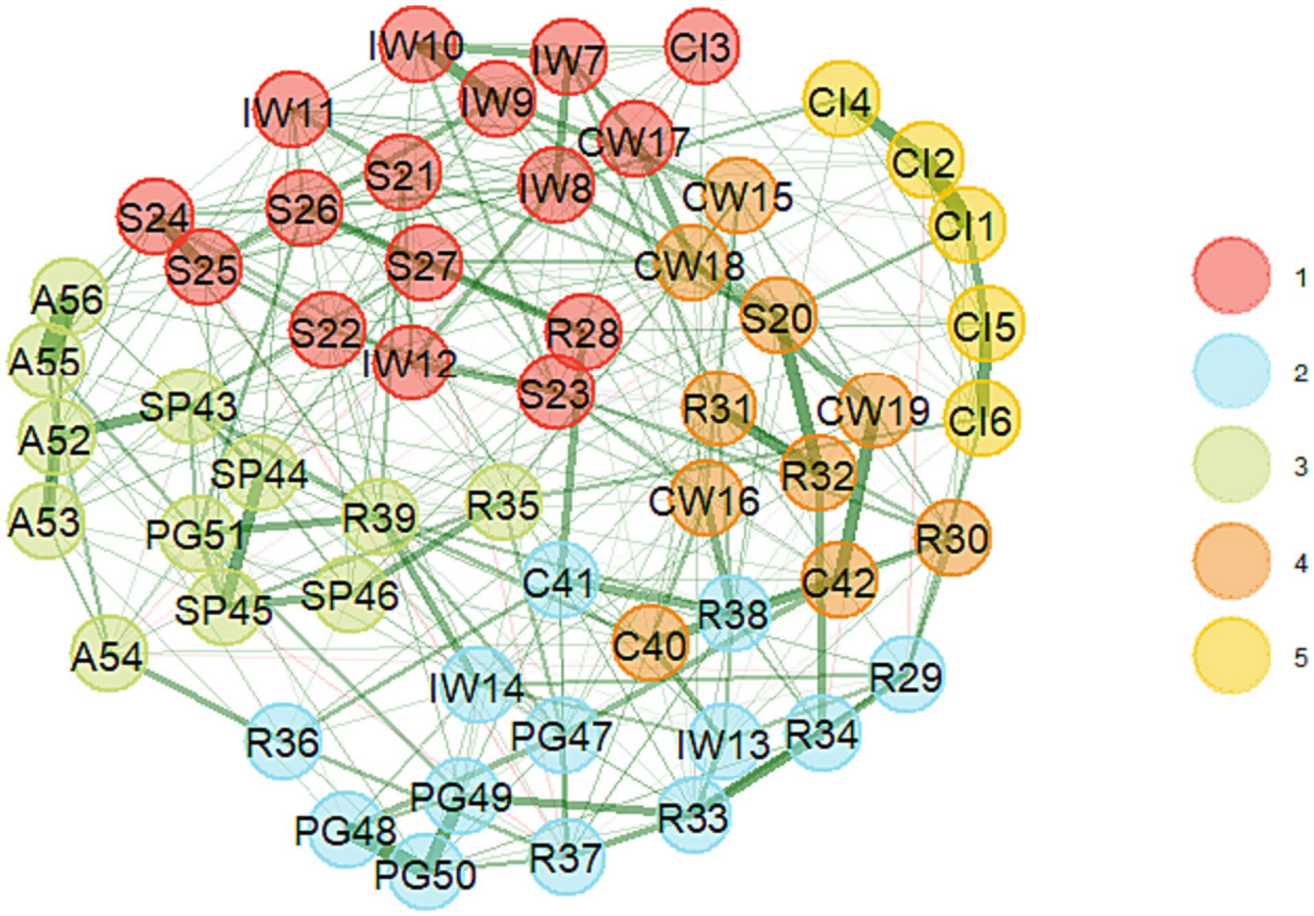
The EGA-network structure (and number of dimensions) of initial (56 items) version of *POS*. Positive edges are displayed as green lines, and negative edges are displayed as red lines. Edge weights are represented by the thickness and saturation of the edges.

Exploratory Graph Analysis (EGA) conducted on the EFA sample suggested the extraction of 5 or 6 clusters in the partial correlation matrix. The results of the dimension stability analysis (based on 1,000 replica samples) confirmed that the five-factor model replicated slightly more frequently than the six-factor model: 36.0% vs. 28.0% of cases. According to the network estimation, the first cluster included 16 items, the second – 13 items, the third – 12 items, the fourth – 10 items and the fifth – 5 items (see Figure 1).

The parallel analysis also suggested the extraction of five factors, which accounted for 59% of the overall variance. Thus, a five-dimensional solution was chosen for the CFA analysis.

The initial five-factor oblimin model (Model 1, see Table 1) had unsatisfactory fit. The factor model was then successively reduced based on the modification indices. In total, 29 items were removed from the questionnaire, having: (a) factor loads less than 0.20 and/or high cross-correlation (8 items); (b) multiple and high covariance of errors among themselves and with other elements (13 items), (c) contradiction with the semantic content of the subscale in which they were included (3 items).

**Table 1:** CFA fit statistics for the tested models of *POS*.

	*χ*^2^ (df)	*p*-value	RMSEA [95% CI]	PCLOSE	SRMR	CFI	TLI	AIC
Model 1: 56 items, five-factor correlated	2817.11 (1474)	<0.001	0.070 [0.066–0.074]	<0.001	0.085	0.767	0.756	29501.10
Model 2: 27 items five-factor correlated	388.63 (314)	0.003	0.036 [0.023–0.046]	0.988	0.054	0.966	0.961	14209.171
Model 3: 27 items, five-factor higher-order	405.139 (319)	0.001	0.038 [0.026–0.048]	0.973	0.058	0.960	0.956	14216.93
Model 4: 27 items five-factor bi-factor	393.560 (297)	0.002	0.037 [0.025–0.047]	0.986	0.055	0.965	0.959	14212.386

**Figure 2:**
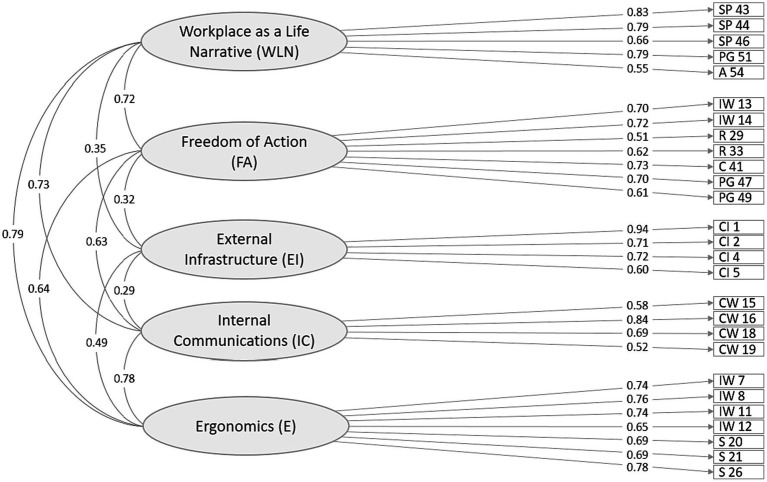
Factor structure of the final version of *POS* (Model 2). Error variances omitted for clarity.

The resulting five-factor oblimin model with 27 items included (Model 2, see Figure 2) had satisfactory fit indices (*χ*^2^ = 388.63, df = 314, *p* = 0.003; RMSEA (0.036) [95% CI, 0.023–0.046]; PCLOSE <0.988, SRMR = 0.054, CFI = 0.966, TLI = 0.961), high factor loadings (0.51–0.94), and acceptable R^2^ values (<0.30) for each item; variances of all items, as well as factors were significant (<0.001). The factors positively correlated with each other in the range from 0.28 to 0.81 (mean correlation = 0.58). As for the semantic content, the items included in each factor obviously reflected the meaning of the construct. Factor 1 (7 items) *Ergonomics (E)* describes facilities that ensure the employee’s comfort and safety in the office. Factor 2 (4 items) *Internal Communications (IC)* implies workplace conditions for effective interaction between employees, and gathering large and small groups. Factor 3 (4 items) *External Infrastructure (EI)* describes the transport accessibility of the area in which the workplace is located and the presence of socially significant objects (cafes, stores, banks). Factor 4 (7 items) *Freedom of Action (FA)* reflects the range of affordances allowing an employee to flexibly adapt their workplace to the current professional tasks and private and social needs, like restoration, hobbies, sports, or communication with family/friends. Factor 5 (5 items) labeled *Workplace as a Life Narrative (WLN)* describes a workplace as an element of place identity that supports the life philosophy of the employee, professional interests, and the history of their career.

We tested which model structure – correlated, high-level, or bi-factor – best described the empirical data. To do this, we built Model 3 including five uncorrelated first-order factors loaded on a higher-order overall factor, as well as a bi-factor model 4 with a general factor and five specific orthogonal factors. Both of these models did not require any major structural modifications but had slightly lower fit than the correlated Model 2 (see Table 1). So, we chose a five-factor correlated Model 2 for further analysis of its psychometric properties.

### 3.3. Measurement invariance and latent means comparison

To test the comparability of the *POS* values and compare the mean of latent variables across different groups, we examined measurement invariance across sex (men, *N* = 98 vs. women, *N* = 221) and age. The criterion for dividing the sample according to the age factor was the respondents’ belonging to generation X (41–60 years; *N* = 136) and generation Y (22–40 years; *N* = 119). The configural model for sex groups together had an adequate fit to the data (*χ*^2^ = 775.810, df = 628.00, *p-*value < 0.001; RMSEA = 0.038, CFI = 0.956, TLI = 0.950). The changes in the fit indices of the metric model indicated no significant decrement in fit from the configural model (*χ*^2^ = 802.91, df = 650, *p-*value < 0.001; ΔCFI = 0.002, ΔTLI = 0, ΔRMSEA = 0). The scalar model had no significant difference in comparison to the metric model (*χ*^2^ = 827.95, df = 672, *p-*value < 0.001; ΔCFI = −0.001, ΔTLI = 0.001, ΔRMSEA = 0), concluding that strong invariance is achieved and the equivalence testing of latent means can be continued. Significant intergroup differences were found on three out of five scales, namely *WLN*, *FA*, and *E* (differences in means: 1.25, 1.89, and 1.57 respectively; *F* = 5.013, *p* = 0.026, *F* = 5.280, *p* = 0.032, *F* = 4.268, *p* = 0.040 respectively; Hedges’ g = 0.27, 0.28, and 0.25 respectively); all indicators are more pronounced in men (see Supplementary Table 3 for descriptive statistics).

The configural model for age groups without any constraints had an adequate fit with incremental indices slightly below the 0.95 cutoff (*χ*^2^ = 822.56, df = 628, *p-*value < 0.001, RMSEA = 0.046, CFI = 0.941, TLI = 0.934). Constraining all factor loadings to be invariant across age samples resulted in a non-significant change in model fit as compared to the baseline model: (*χ*^2^ = 848.81, df = 650, *p-*value < 0.001; ΔCFI = −0.001, ΔTLI = 0.001, ΔRMSEA = 0). Constraining all intercepts to be invariant did not lead to a significant worsening of model fit: *χ*^2^ = 887.96, df = 672, *p-*value < 0.001; ΔCFI = −0.005, ΔTLI = 0.003, ΔRMSEA = 0.001. A comparative analysis of the latent means of the *POS* factors in young people and adults did not reveal significant differences in any of the scales (*p* > 0.05).

### 3.4. Internal reliability and construct validity

To assess the internal reliability of *POS* on the CFA sample data, we used McDonald’s omega coefficients (ω). All five scales had satisfactory reliability coefficients (95% confidence intervals are in square brackets): ω = 0.85 [0.82–0.89] for *WLN* scale; ω = 0.84 [0.80–0.87] for *FA;* ω = 0.83 [0.78–0.88] for *EI*; ω = 0.77 [0.70–0.82] for *IC*; and ω = 0.88 [0.86–0.91] – for *E* scale. The internal convergent validity of the scales has been checked on the CFA sample by verifying that the average variance extracted (AVE) values were greater than 0.5 (Fornell and Larcker, 1981). The *WLN*, *EI*, and *E* subscales reached the AVE threshold (0.55, 0.56, and 0.53 respectively), while the extracted variance of the *FA* and *IC* scales were below the target values (AVE = 0.44 and 0.46 respectively). To ensure the discriminant validity of the scales, we compared the square root of the AVE to inter-construct correlations: if the levels of the square root of the AVE for each scale is greater than inter-construct correlations (ICC), the discriminant validity is supported. A lack of discriminant validity was found comparing for 6 pairs of constructs out of 20: *WLN–E* (√AVE = 0.74 < ICC = 0.79), *FA–WLN* (√AVE = 0.66 < ICC = 0.72), *IC–WLN* (√AVE = 0.68 < ICC = 0.73), *IC–E* (√AVE = 0.68 < ICC = 0.78), *E–WLN* (√AVE = 0.73 < ICC = 0.79), and *E–IC* (√AVE = 0.73 < ICC = 0.78). Thus, the *FA, IC,* and *E* scales are the worst differentiated.

When testing external convergent and divergent validity, well-interpreted results were obtained, namely, convergent measures (*OAS*, *PRS*, and *OCS*) moderately correlated with the subscales of *POS* (mean correlations = 0.543, 0.401, and −0.227, respectively), while the divergent measure (*WEMWBS*) had weak correlations (mean correlations = 0.175; see Supplementary Table 4). The strongest associations were found between the *WLN* and *E* scales and the *OAS*, as well as between *WLN* and *Compatibility* from *PRS*. Subscales of *OCS* correlated weaker than other convergent measures with the *POS* subscales; all of them, as expected, had negative associations.

## 4. Discussion

This research aimed to develop a new standardized method for assessing healthy workplace environments using positive psychology, salutogenic, and biophilic design. As far as we know, such methods have not been developed before, which caused the topicality and novelty of the study. We have achieved this goal: A new instrument called the *People in the Office Scale (POS)* has been developed. It comprises 27 items across five subscales: *Ergonomics, Internal Communications, External Infrastructure, Freedom of Action*, and *Workplace as a Life Narrative* which are easily interpreted and correspond well to the multidimensional needs of employees satisfied with their office environment (Altomonte et al., 2020; Bluyssen, 2022). *POS* demonstrates high structural and substantive validity, along with internal reliability. This scale addresses a significant gap in the field of organizational psychology by enabling a universal multifactorial assessment of work environments in offices of various types, and in conditions of remote work or face-to-face presence. It complements existing tools (Chau et al., 2012; Duncan et al., 2013; Jancey et al., 2014; Scrima, 2015; Alonso Nuez et al., 2022), which have primarily focused on employees’ activities as, in contrast, *POS* allows for assessing the work environment as it is perceived and utilized by the employees. Compared to other instruments that measure social relations in the workplace (Razak et al., 2016; Pacheco and Coello-Montecel, 2020), *POS* is specifically designed to consider a specialist as an agent of their professional life (Deci and Ryan, 2008) and assess individual affordances that contribute to personal well-being. As such, it is instrumental in identifying opportunities for modifying and enhancing these environments as needed.

While examining measurement invariance across sex and age, the configural, metric, and scalar invariances of the model were achieved. Latent mean comparison in sex subgroups revealed intergroup differences in *WLN, FA*, and *E* scores which are more pronounced in men. Age-specific differences were not found for any of the subscales.

The internal convergent validity (as measured by AVE) was confirmed for the *WLN, EI*, and *E* but not for the *FA* and *IC* subscales. The external convergent validity check showed satisfactory results: as expected, all the scores of the newly developed scale positively correlated with the scores of the *Office Attachment Scale,* the *Perceived Restorativeness Scale*, and negatively with the *Organizational Cynicism Scale*. The external divergent validity check was also successful: in accordance with the meaning of the correlated variables, there were weak connections between mental well-being and *POS*. But in terms of internal divergent validity the *FA, IC*, and *E* scales demonstrated a lack of discriminant validity.

*POS* subscales present various human needs, starting with a need for security *(Ergonomics)* and finishing with the needs for identity and self-realization *(Workplace as a Life Narrative).* To sum up, *POS* can be recommended both for researchers and practitioners, like organization leaders and managers, who are interested in a healthy and user-friendly office atmosphere, employee’s psychological well-being, loyalty to their organization, quality of life, etc.

## 5. Limitations and prospects

In the future studies, we plan to expand our sample by representing more diverse professions, to use a wider range of variables for additional verification of convergent and divergent validity, to arrange examination of the tool in more controlled office conditions, and, as we hope that the method can be used outside of Russia, to examine its cross-cultural measurement invariance.

## Data availability statement

The raw data supporting the conclusions of this article will be made available by the authors, without undue reservation.

## Ethics statement

The studies involving human participants were reviewed and approved by the Commission for the Ethical Evaluation of Empirical Research Projects of the Department of Psychology at HSE University. The patients/participants provided their written informed consent to participate in this study and publish this data anonymously.

## Author contributions

MP collected data, organized the database, wrote the first draft of the manuscript, and contributed to the manuscript revision. SR contributed to the study’s conception and design, performed the statistical analyses, and contributed to the manuscript revision. SN-B developed the main idea of the manuscript, wrote the first draft of the manuscript, and contributed to the manuscript revision. All authors contributed to the article and approved the submitted version.

## References

[ref1] Al-Dmour Y. Garaj V. Clements-Croome D. (2021). The flourishing of Biophilic workplaces: ‘second home’ offices as a case study. Intellig. Build. Int. 13, 261–274. doi: 10.1080/17508975.2020.1807895

[ref2] Allen J.G. Bernstein A. Cao X. Eitland E.S. Flanigan S. Gokhale M.. (2017). The 9 foundations of a healthy building. Harvard T.H. Chan School of Public Health, Center for Health and Global Environment.

[ref3] Alonso Nuez M. J. Gil Lacruz M. García Madurga M. Á. Lairla C. Saz Gil M. I. Rosell Martínez J.. (2022). Corporate social responsibility and workplace health promotion: a systematic review. Front. Psychol. 13:1011879. doi: 10.3389/fpsyg.2022.1011879, PMID: 36312126 PMC9610113

[ref4] Altomonte S. Allen J. Bluyssen P. M. Brager G. Heschong L. Loder A.. (2020). Ten questions concerning well-being in the built environment. Build. Environ. 180:106949. doi: 10.1016/j.buildenv.2020.106949

[ref5] Antonosky A. (1996). The salutogenetic model as a theory to guide health promotion. Health Promot. Int. 11, 11–18. doi: 10.1093/heapro/11.1.11

[ref6] Armitage L. A. Nassor Amar J. H. (2021). “Person-environment fit theory: application to the design of work environments” in A handbook of theories on designing alignment between people and the office environment. eds. Appel-Meulenbroek R. Danivska V. (London: Routledge), 14–26.

[ref7] Banga A. Mahajan F. (2021). Work from home and changing dynamics. J. Manag. Res. Anal. 8, 78–88. doi: 10.18231/j.jmra.2021.017

[ref8] Bauer A. C. (2020). Pride and productivity – introducing and testing the healing offices® design concept. Psychol. J. Corp. Real Estate 22, 313–340. doi: 10.1108/JCRE-02-2019-0012

[ref9] Bergefurt L. Weijs-Perrée M. Appel-Meulenbroek R. Arentze T. (2022). The physical office workplace as a resource for mental health– A systematic scoping review. Build. Environ. 207:108505. doi: 10.1016/j.buildenv.2021.108505

[ref10] Bluyssen P. (2022). Patterns and profiles for understanding the indoor environment and its occupants. CLIMA 2022 Conference.

[ref11] Brandes P. Dharwadkar R. Dean J. (1999). Does organizational cynicism matter? Employee and Supervisior perspectives on work outcomes. The 36th annual meeting of the eastern academy of management, Philadelphia, 1–33.

[ref12] Browning B. Cooper C. (2016). The global impact of biophilic design in the workplace. ArchitectureNow, New Zealand. Available at: https://architecturenow.co.nz/articles/the-global-impact-of-biophilic-design-in-the-workplace/ (Accessed August 22, 2023).

[ref13] Browning W. Ryan C. Clancy J. (2014). 14 patterns of biophilic design improving health & well-being in the built environment. Available at: https://www.terrapinbrightgreen.com/wp-content/uploads/2014/09/14-Patterns-of-Biophilic-Design-Terrapin-2014p.pdf (Accessed August 22, 2023).

[ref14] Burton E. Weich S. Blanchard M. Prince M. (2005). Measuring physical characteristics of housing: the built environment site survey checklist (BESSC). Environ. Plan. Plan. Design 32, 265–280. doi: 10.1068/b3038

[ref15] Chau J. Y. Van Der Ploeg H. P. Dunn S. Kurko J. Bauman A. E. (2012). Validity of the occupational sitting and physical activity questionnaire. Med. Sci. Sports Exerc. 44, 118–125. doi: 10.1249/MSS.0b013e3182251060 21659903

[ref16] Chen F. F. (2007). Sensitivity of goodness of fit indexes to lack of measurement invariance. Struct. Equat. Model. A Multidiscipl. J. 14, 464–504. doi: 10.1080/10705510701301834

[ref17] Clements-Croome D. Turner B. Pallaris K. (2019). Flourishing workplaces: a multisensory approach to design and POE. Intellig. Build. Int. 11, 131–144. doi: 10.1080/17508975.2019.1569491

[ref18] Cohen J. (1988). Statistical power analysis for the behavioral sciences, 2nd Edn. Hillsdale, NJ: Lawrence Erlbaum Associates.

[ref19] Colenberg S. Jylhä T. Arkesteijn M. (2021). The relationship between interior office space and employee health and well-being–a literature review. Build. Res. Inf. 49, 352–366. doi: 10.1080/09613218.2019.1710098

[ref20] Damaske S. Smyth J. M. Zawadzki M. J. (2014). Has work replaced home as a haven? Reexamining Arlie Hochschild’s time bind proposition with objective stress data. Soc. Sci. Med. 115, 130–138. doi: 10.1016/j.socscimed.2014.04.047, PMID: 24869785 PMC4209911

[ref21] De Cooman R. Vleugels W. (2022). “Person–environment fit: theoretical perspectives, conceptualizations, and outcomes”. Oxford research encyclopedia of business and management. Available at: https://oxfordre.com/business/view/10.1093/acrefore/9780190224851.001.0001/acrefore-9780190224851-e-377 (Accessed August 22, 2023).

[ref22] Deci E. L. Ryan R. M. (2008). Self-determination theory: a macrotheory of human motivation, development, and health. Can. Psychol. 49, 182–185. doi: 10.1037/a0012801

[ref23] Dilani А. (2008). Psychosocially supportive design: a salutogenic approach to the design of the physical environment. Available at: https://www.researchgate.net/publication/265349464_Psychosocially_Supportive_Design_A_Salutogenic_Approach_to_the_Design_of_the_Physical_Environment (Accessed August 22, 2023).

[ref24] Duncan M. J. Rashid M. Vandelanotte C. Cutumisu N. Plotnikoff R. C. (2013). Development and reliability testing of a self-report instrument to measure the office layout as a correlate of occupational sitting. Int. J. Behav. Nutr. Phys. Act. 10, 16–12. doi: 10.1186/1479-5868-10-16, PMID: 23379485 PMC3576330

[ref25] Edwards J. Caplan R. D. Harrison R. V. (1998). “Person-environment fit theory: conceptual foundations, empirical evidence, and directions for future research” in Theories of organizational stress. ed. Cooper C. L. (Oxford: Oxford University Press), 28–67.

[ref26] Fischer R. Karl J. A. (2019). A primer to (cross-cultural) multi-group invariance testing possibilities. Front. Psychol. 10:1507. doi: 10.3389/fpsyg.2019.01507, PMID: 31379641 PMC6657455

[ref27] Fornell C. Larcker D. F. (1981). Evaluating structural equation models with unobservable variables and measurement error. J. Mark. Res. 18, 39–50. doi: 10.2307/3151312

[ref28] Forooraghi M. Cobaleda-Cordero A. Chafi M. B. (2022). A healthy office and healthy employees: a longitudinal case study with a salutogenic perspective in the context of the physical office environment. Build. Res. Inf. 50, 134–151. doi: 10.1080/09613218.2021.1983753

[ref29] Golembiewski J. A. (2016). The impact of workplace design on mental wellbeing: Discoveries and future directions. Conference: The Workplace Health Promotion Network Annual Forum. Available at: https://www.researchgate.net/publication/304254826_The_impact_of_workplace_design_on_mental_wellbeing_discoveries_and_future_directions (Accessed August 22, 2023).

[ref30] Golembiewski J. A. (2022). “Salutogenic Architecture” in The handbook of Salutogenesis. eds. Mittelmark M. B.., 259–274. doi: 10.1007/978-3-030-79515-3_26

[ref31] Golino H. Christensen A. (2022). EGAnet: Usefu exploratory graph analysis – a framework for estimating the number of dimensions in multivariate data using network psychometrics. R Package Version 1.2.3. Available at: https://cran.r-project.org/web/packages/EGAnet/EGAnet.pdf (Accessed January 6, 2022).

[ref32] Gönülateş E. (2019). Quality of item Pool (QIP) index: A novel approach to evaluating CAT item Pool adequacy. Educ. Psychol. Meas. 79, 1133–1155. doi: 10.1177/0013164419842215, PMID: 31619842 PMC6777062

[ref33] Gravetter F. Wallnau L. (2014). Essentials of statistics for the behavioral sciences (8th Edition). Belmont, CA: Wadsworth.

[ref34] Haapakangas A. Sirola P. Ruohomaki V. (2022). Workspace use, perceived work environment and employee wellbeing – A case study of an activity-based office. 51st Nordic ergonomics and human factors society conference. Available at: https://www.researchgate.net/profile/Cecilia-Osterman/publication/364647449_NES2022_WORK_WELL_Conference_Proceedings/links/6355076796e83c26eb45c979/NES2022-WORK-WELL-Conference-Proceedings.pdf#page=108 (Accessed August 22, 2023).

[ref35] Hair J. F. Black W. C. Babin B. J. Anderson R. E. (2010). Multivariate data analysis: A global perspective. Hoboken, NJ: Pearson Prentice Hall.

[ref36] Hartig T. Korpela K. Evans G. W. Gärling T. (1997). A measure of restorative quality in environments. Scandinavian Hous. Plan. Res. 14, 175–194. doi: 10.1080/02815739708730435

[ref37] Heerwagen J. H. Haubach J. G. Montgomery J. Weimer W. C. (1995). Environmental design, work, and well-being: managing occupational stress through changes in workplace environment. Official J. Am. Assoc. Occup. Health Nurs. 43, 458–468. 7545995

[ref38] Hochschild A. R. (2003). The commercialization of intimate life: Notes from home and work. Berkeley: University of California Press.

[ref39] Hu L. T. Bentler P. M. (1999). Cutoff criteria for fit indexes in covariance structure analysis: conventional criteria versus new alternatives. Struct. Equ. Model. 6, 1–55. doi: 10.1080/10705519909540118

[ref40] Jancey J. Tye M. McGann S. Blackford K. Lee A. H. (2014). Application of the occupational sitting and physical activity questionnaire (OSPAQ) to office-based workers. BMC Public Health 14, 1–6. doi: 10.1186/1471-2458-14-762 25069528 PMC4132919

[ref41] Jorgensen T. D. Pornprasertmanit S. Schoemann A. M. Rosseel Y. (2022). SemTools: useful tools for structural equation modeling. R package version 0.5-6. Available at: https://cran.r-project.org/web/packages/semTools/semTools.pdf (Accessed January 6, 2022).

[ref42] Kaplan S. (1995). The restorative benefits of nature: toward an integrative framework. J. Environ. Psychol. 15, 169–182. doi: 10.1016/0272-4944(95)90001-2

[ref43] Kartik A. Neeraj R. (2013). Itemized analysis of questions of multiple choice question (MCQ) exam. IJSR 2, 279–280. doi: 10.36106/IJSR

[ref44] Kellert S. Calabrese E. (2015). The practice of Biophilic design. Available at: https://www.biophilic-design.com/ (Accessed August 22, 2023).

[ref45] Kelly L. Jenkinson C. Ziebland S. (2013). Measuring the effects of online health information for patients: item generation for an e-health impact questionnaire. Patient Educ. Couns. 93, 433–438. doi: 10.1016/j.pec.2013.03.012, PMID: 23598293 PMC3863952

[ref46] Mura A. L. Ariccio S. Villani T. Bonaiuto F. Bonaiuto M. (2023). The physical environment in remote working: development and validation of perceived remote workplace environment quality indicators (PRWEQIs). Sustainability 15:2858. doi: 10.3390/su15042858

[ref47] Pacheco P. O. Coello-Montecel D. (2020). The working conditions questionnaire: cross-cultural validation and scale refinement in six Ibero-American countries. Empl. Relat. 43, 1016–1028. doi: 10.1108/ER-05-2020-0240

[ref48] Pavlova M. V. Dzyubenko M. M. Nartova-Bochaver S. K. (2022). The organizational cynicism scale: an adaptation on the Russian-speaking sample. Soc. Psychol. Soc. 13, 184–200. doi: 10.17759/sps.2022130311

[ref49] Pavlova M. V. Nartova-Bochaver S. K. (2020). Routine self-help behaviors of employees (in case of architect offices). Organ. Psychol. 10, 164–184.

[ref50] Polit D. F. Beck C. T. (2006). The content validity index: are you sure you know what’s being reported? Critique and recommendations. Res. Nurs. Health 29, 489–497. doi: 10.1002/nur.20147 16977646

[ref51] R Core Team (2022). R: A language and environment for statistical computing. R foundation for statistical computing, Vienna, Austria. Available at: https://www.r-project.org/ (Accessed January 6, 2022).

[ref52] Rasheed E. O. Rotimi J. O. B. (2022). The green office environment: New Zealand workers' perception of IEQ. Smart Sustain. Built Environ. doi: 10.1108/SASBE-09-2022-0204

[ref53] Razak N. A. Ma’amor H. Hassan N. (2016). Measuring reliability and validity instruments of work environment towards quality work life. Proc. Eco. Fin. 37, 520–528. doi: 10.1016/S2212-5671(16)30160-5

[ref54] Revelle W. (2022). Psych: procedures for psychological, psychometric, and personality research. R package version 2.2.9. Available at: https://cran.r-project.org/web/packages/psych/psych.pdf (Accessed January 6, 2022).

[ref55] Robinson O. C. Lopez F. G. Ramos K. Nartova-Bochaver S. (2013). Authenticity, social context, and well-being in the United States, England, and Russia: A three country comparative analysis. J. Cross-Cult. Psychol. 44, 719–737. doi: 10.1177/0022022112465672

[ref56] Roskams M. Haynes B. (2019). Salutogenic workplace design: a conceptual framework for supporting sense of coherence through environmental resources. J. Corp. Real Estate 22, 139–153. doi: 10.1108/JCRE-01-2019-0001

[ref57] Rosseel Y. (2022). lavaan: latent variable analysis. An R package for structural equation modeling. R Package Version 0.6–12. Available at: https://cran.r-project.org/web/packages/lavaan/lavaan.pdf (Accessed January 6, 2022).

[ref58] Scrima F. (2015). The convergent-discriminant validity of the workplace attachment scale (WAS). J. Environ. Psychol. 43, 24–29. doi: 10.1016/j.jenvp.2015.05.009

[ref59] Tennant R. Hiller L. Fishwick R. Platt S. Joseph S. Weich S.. (2007). The Warwick-Edinburgh mental well-being scale (WEMWBS): development and UK validation. Health Qual. Life Outcomes 5:63. doi: 10.1186/1477-7525-5-63 18042300 PMC2222612

[ref60] Timm S. Gray W. Curtis T. Chung S. (2018). Designing for health: how the physical environment plays a role in workplace wellness. Am. J. Health Promot. 32, 1468–1473. doi: 10.1177/0890117118779463b 29972070

[ref61] Vandelannoitte A. L. (2021). The new paternalism? The workplace as a place to work-and to live. Organization 28, 949–975. doi: 10.1177/13505084211015374.hal-03328163

[ref62] Vischer J. C. (2008). Towards an environmental psychology of workspace: how people are affected by environments for work. Archit. Sci. Rev. 51, 97–108. doi: 10.3763/asre.2008.5114

[ref63] Wild D. Grove A. Martin M. Eremenco S. McElroy S. Verjee-Lorenz A.. (2005). Principles of good practice for the translation and cultural adaptation process for patient-reported outcomes (PRO) measures: report of the ISPOR task force for translation and cultural adaptation. Value Health 8, 94–104. doi: 10.1111/j.1524-4733.2005.04054.x, PMID: 15804318

[ref64] Williams D. R. Vaske J. J. (2003). The measurement of place attachment: validity and generalizability of a psychometric approach. For. Sci. 49, 830–840.

[ref65] World Health Organization Burton J. (2010). WHO healthy workplace framework and model: background document and supporting literature and practices. World Health Organization. Available at: https://apps.who.int/iris/handle/10665/113144 (Accessed April 12, 2023).

