# Supplementary material for: A new instrument to measure healthy workplace qualities: the People in the Office Scale
Source: Front Psychol. 2023 Nov 2;14:1241555. doi: 10.3389/fpsyg.2023.1241555 (PMC10658938; doi:10.3389/fpsyg.2023.1241555)
Supplement: Supplementary file 1 [file Table_1.docx]

Appendix 1

Initial and final lists of the items: a form of the *People in the Office* scale^[[1]](#footnote-1)^

Instruction.

Dear participant!

Please observe or visualize your current workplace and tick the box to what extent the statements below describe its properties. By "office," we mean a workspace which can vary depending on your profession – for some it may be a school, for others a hospital or a company specializing in fields such as pharmaceuticals, IT, or other professions.

Thank you for your participation!

| #  Initial | #  New | Scale | Statement | Strongly disagree | Rather disagree | Hard to answer | Rather agree | Strongly agree |
| --- | --- | --- | --- | --- | --- | --- | --- | --- |
|  |  |  |  | 1 | 2 | 3 | 4 | 5 |
| 1 | 13 | *EI* | The office is located in an attractive area of the city (clean, safe, and active). |  |  |  |  |  |
| 2 | 15 | *EI* | The office is surrounded by convenient infrastructure, including cafes, shops, and banks. |  |  |  |  |  |
| 3 |  | deleted | The office has convenient parking. |  |  |  |  |  |
| 4 | 12 | *EI* | The office is easily accessible via public transportation. |  |  |  |  |  |
| 5 | 14 | *EI* | There are walking areas near the office. |  |  |  |  |  |
| 6 |  | deleted | There is a sports facility near the office. |  |  |  |  |  |
| 7 | 6 | *E* | The office provides the employee with an ergonomic workplace. |  |  |  |  |  |
| 8 | 3 | *E* | Office space is comfortable for work (lighting, acoustics, temperature, air and water quality, smell, and safety). |  |  |  |  |  |
| 9 |  | deleted | The office is equipped with modern technologies and equipment. |  |  |  |  |  |
| 10 |  | deleted | There is an efficient storage system for paperwork and tools, encompassing everything needed for work. |  |  |  |  |  |
| 11 | 4 | *E* | There is a convenient storage system for personal belongings like coats, shoes, etc. |  |  |  |  |  |
| 12 | 5 | *E* | The workspace is conducive to focusing on tasks. |  |  |  |  |  |
| 13 | 20 | *FA* | Employees have the flexibility to choose their workspace every day based on their mood, state, and tasks. |  |  |  |  |  |
| 14 | 17 | *FA* | The office allows employees customizing workspaces according to their needs. |  |  |  |  |  |
| 15 | 10 | *IC* | The layout facilitates easy communication among colleagues throughout the work process. |  |  |  |  |  |
| 16 | 9 | *IC* | There are quiet spaces for small group discussions. |  |  |  |  |  |
| 17 |  | deleted | The office layout is easy to navigate and allows for optimal mobility. |  |  |  |  |  |
| 18 | 11 | *IC* | The office design allows employees to either observe or participate in large events. |  |  |  |  |  |
| 19 | 8 | *IC* | Office space allows staff to gather the team when necessary. |  |  |  |  |  |
| 20 | 1 | *E* | Office space is well-planned with easily accessible amenities such as toilets, kitchen, etc. |  |  |  |  |  |
| 21 | 7 | *E* | The office layout respects and defines employees' personal space. |  |  |  |  |  |
| 22 |  | deleted | Office space is divided into zones for different activities. |  |  |  |  |  |
| 23 |  | deleted | The office is spacious, allowing for comfortable distancing. |  |  |  |  |  |
| 24 |  | deleted | Office space gives the staff an opportunity to feel in "their own" territory. |  |  |  |  |  |
| 25 |  | deleted | Office space conveys a sense of stability. |  |  |  |  |  |
| 26 | 2 | *E* | Office space is aesthetically pleasing, clean, and well-maintained |  |  |  |  |  |
| 27 |  | deleted | Office space engages the senses through auditory, visual, and olfactory stimuli. |  |  |  |  |  |
| 28 |  | deleted | Office space allows the staff to take care of themselves when they need it. |  |  |  |  |  |
| 29 | 19 | *FA* | The office includes gym equipment for physical exercise. |  |  |  |  |  |
| 30 |  | deleted | There is room for movement and stretching. |  |  |  |  |  |
| 31 |  | deleted | Office space provides an opportunity to drink tea or coffee. |  |  |  |  |  |
| 32 |  | deleted | A kitchen is accessible for meals. |  |  |  |  |  |
| 33 | 22 | *FA* | There are areas for relaxation and sleep if needed. |  |  |  |  |  |
| 34 |  | deleted | Shower facilities are available. |  |  |  |  |  |
| 35 |  | deleted | Office spaces give people the opportunity to look out the window into the distance. |  |  |  |  |  |
| 36 |  | deleted | Office space allows people to grow their favorite plants if they wish. |  |  |  |  |  |
| 37 |  | deleted | The office space is pet-friendly. |  |  |  |  |  |
| 38 |  | deleted | There are quiet areas for privacy and relaxation. |  |  |  |  |  |
| 39 |  | deleted | Office space offers a valuable opportunity to cultivate strength and find inspiration. |  |  |  |  |  |
| 40 |  | deleted | Office space can accommodate guests. |  |  |  |  |  |
| 41 | 18 | *FA* | The office accommodates communication with family members or children when needed. |  |  |  |  |  |
| 42 |  | deleted | The office space is suitable for social and cultural events. |  |  |  |  |  |
| 43 | 26 | *WLN* | My workplace reflects my life philosophy. |  |  |  |  |  |
| 44 | 25 | *WLN* | Office space represents the professional interests, achievements, and victories of employees. |  |  |  |  |  |
| 45 |  | deleted | Office space reflects the professional status of the employees. |  |  |  |  |  |
| 46 | 24 | *WLN* | Office space can store the historical records and achievements of the business. |  |  |  |  |  |
| 47 | 21 | *FA* | The office provides space for pursuing hobbies. |  |  |  |  |  |
| 48 |  | deleted | Office space lets people listen to music if they want to. |  |  |  |  |  |
| 49 | 16 | *FA* | The office gives space for reading and relaxation. |  |  |  |  |  |
| 50 |  | deleted | There are facilities for watching movies or listening to podcasts. |  |  |  |  |  |
| 51 | 23 | *WLN* | Office space reflects and reminds employees of their dreams. |  |  |  |  |  |
| 52 |  | deleted | I feel like my workplace is a part of me. |  |  |  |  |  |
| 53 |  | deleted | Those who see my workplace can learn a lot about me. |  |  |  |  |  |
| 54 | 27 | *WLN* | In the context of lifestyle and outlook, I find that I am akin to those who are employed in my workplace. |  |  |  |  |  |
| 55 |  | deleted | I love working at my workplace. |  |  |  |  |  |
| 56 |  | deleted | I like my workplace. |  |  |  |  |  |

*Notes*. *E* – *Ergonomics*, *IC* - *Internal communication*, *EI* – *External infrastructure*, *FA* – *Freedom of action, WLN* – *Work as a life narrative;* deleted - the item was deleted after the full psychometric examination.

1. To get a convenient form of the tool, please contact the authors. [↑](#footnote-ref-1)
