# Supplementary material for: A new instrument to measure healthy workplace qualities: the People in the Office Scale
Source: Front Psychol. 2023 Nov 2;14:1241555. doi: 10.3389/fpsyg.2023.1241555 (PMC10658938; doi:10.3389/fpsyg.2023.1241555)
Supplement: Supplementary file 2 [file Table_2.docx]

Appendix 2

Descriptive statistics and parameters of item analysis

| # | Item | Mean | SD | Med | Min | Max | 25th | 75th | Sk | Ku | Diff | Disc | ITC | Alpha, drop |
| --- | --- | --- | --- | --- | --- | --- | --- | --- | --- | --- | --- | --- | --- | --- |
| 1 | The office is located in an attractive area of the city (clean, safe, and active). | 3.88 | 1.19 | 4.00 | 1.00 | 5.00 | 4.00 | 5.00 | -1.05 | 0.18 | 0.69 | 0.26 | 0.41 | 0.964 |
| 2 | The office is surrounded by convenient infrastructure, including cafes, shops, and banks. | 3.95 | 1.22 | 4.00 | 1.00 | 5.00 | 4.00 | 5.00 | -1.02 | -0.15 | 0.74 | 0.22 | 0.34 | 0.964 |
| 3 | The office has convenient parking. | 3.30 | 1.38 | 4.00 | 1.00 | 5.00 | 2.00 | 4.00 | -0.33 | -1.18 | 0.58 | 0.23 | 0.32 | 0.965 |
| 4 | The office is easily accessible via public transportation. | 3.91 | 1.20 | 4.00 | 1.00 | 5.00 | 4.00 | 5.00 | -1.02 | -0.02 | 0.73 | 0.27 | 0.40 | 0.964 |
| 5 | There are walking areas near the office. | 3.73 | 1.38 | 4.00 | 1.00 | 5.00 | 2.00 | 5.00 | -0.73 | -0.88 | 0.68 | 0.27 | 0.34 | 0.964 |
| 6 | There is a sports facility near the office. | 3.23 | 1.37 | 3.00 | 1.00 | 5.00 | 2.00 | 4.00 | -0.15 | -1.28 | 0.56 | 0.31 | 0.40 | 0.964 |
| 7 | The office provides the employee with an ergonomic workplace. | 3.75 | 1.14 | 4.00 | 1.00 | 5.00 | 3.00 | 5.00 | -0.87 | -0.08 | 0.69 | 0.43 | 0.69 | 0.963 |
| 8 | Office space is comfortable for work (lighting, acoustics, temperature, air and water quality, smell, and safety). | 3.62 | 1.21 | 4.00 | 1.00 | 5.00 | 2.00 | 5.00 | -0.65 | -0.74 | 0.66 | 0.47 | 0.69 | 0.963 |
| 9 | The office is equipped with modern technologies and equipment. | 3.88 | 1.13 | 4.00 | 1.00 | 5.00 | 4.00 | 5.00 | -0.99 | 0.06 | 0.72 | 0.35 | 0.58 | 0.964 |
| 10 | There is an efficient storage system for paperwork and tools, encompassing everything needed for work. | 3.72 | 1.19 | 4.00 | 1.00 | 5.00 | 3.00 | 5.00 | -0.67 | -0.68 | 0.68 | 0.39 | 0.59 | 0.963 |
| 11 | There is a convenient storage system for personal belongings like coats, shoes, etc. | 3.72 | 1.16 | 4.00 | 1.00 | 5.00 | 3.00 | 5.00 | -0.68 | -0.64 | 0.68 | 0.40 | 0.67 | 0.963 |
| 12 | The workspace is conducive to focusing on tasks. | 3.68 | 1.11 | 4.00 | 1.00 | 5.00 | 3.00 | 4.00 | -0.63 | -0.51 | 0.67 | 0.40 | 0.64 | 0.963 |
| 13 | Employees have the flexibility to choose their workspace every day based on their mood, state, and tasks. | 2.30 | 1.39 | 2.00 | 1.00 | 5.00 | 1.00 | 3.00 | 0.70 | -0.90 | 0.32 | 0.47 | 0.62 | 0.963 |
| 14 | The office allows employees customizing workspaces according to their needs. | 2.90 | 1.32 | 3.00 | 1.00 | 5.00 | 2.00 | 4.00 | -0.01 | -1.28 | 0.47 | 0.45 | 0.64 | 0.963 |
| 15 | The layout facilitates easy communication among colleagues throughout the work process. | 3.94 | 0.99 | 4.00 | 1.00 | 5.00 | 4.00 | 5.00 | -1.13 | 1.06 | 0.74 | 0.28 | 0.50 | 0.964 |
| 16 | There are quiet spaces for small group discussions. | 3.47 | 1.32 | 4.00 | 1.00 | 5.00 | 2.00 | 5.00 | -0.41 | -1.16 | 0.62 | 0.49 | 0.64 | 0.963 |
| 17 | The office layout is easy to navigate and allows for optimal mobility. | 3.74 | 1.09 | 4.00 | 1.00 | 5.00 | 3.00 | 5.00 | -0.69 | -0.37 | 0.68 | 0.40 | 0.63 | 0.963 |
| 18 | The office design allows employees to either observe or participate in large events. | 3.41 | 1.20 | 4.00 | 1.00 | 5.00 | 2.00 | 4.00 | -0.35 | -0.87 | 0.60 | 0.37 | 0.55 | 0.964 |
| 19 | Office space allows staff to gather the team when necessary. | 3.93 | 1.15 | 4.00 | 1.00 | 5.00 | 4.00 | 5.00 | -1.05 | 0.22 | 0.73 | 0.31 | 0.46 | 0.964 |
| 20 | Office space is well-planned with easily accessible amenities such as toilets, kitchen, etc. | 3.78 | 1.19 | 4.00 | 1.00 | 5.00 | 3.00 | 5.00 | -0.86 | -0.33 | 0.69 | 0.41 | 0.65 | 0.963 |
| 21 | The office layout respects and defines employees' personal space. | 3.21 | 1.26 | 4.00 | 1.00 | 5.00 | 2.00 | 4.00 | -0.15 | -1.24 | 0.55 | 0.44 | 0.64 | 0.963 |
| 22 | Office space is divided into zones for different activities. | 3.23 | 1.21 | 3.00 | 1.00 | 5.00 | 2.00 | 4.00 | -0.24 | -1.02 | 0.56 | 0.41 | 0.62 | 0.963 |
| 23 | The office is spacious, allowing for comfortable distancing. | 3.53 | 1.24 | 4.00 | 1.00 | 5.00 | 2.00 | 4.00 | -0.59 | -0.78 | 0.63 | 0.43 | 0.66 | 0.963 |
| 24 | Office space gives the staff an opportunity to feel in "their own" territory. | 3.61 | 1.19 | 4.00 | 1.00 | 5.00 | 3.00 | 4.00 | -0.62 | -0.60 | 0.65 | 0.46 | 0.74 | 0.963 |
| 25 | Office space conveys a sense of stability. | 3.68 | 1.04 | 4.00 | 1.00 | 5.00 | 3.00 | 4.00 | -0.67 | -0.11 | 0.67 | 0.37 | 0.70 | 0.963 |
| 26 | Office space is aesthetically pleasing, clean, and well-maintained | 3.81 | 1.07 | 4.00 | 1.00 | 5.00 | 3.00 | 5.00 | -0.90 | 0.19 | 0.70 | 0.40 | 0.70 | 0.963 |
| 27 | Office space engages the senses through auditory, visual, and olfactory stimuli. | 3.53 | 1.06 | 4.00 | 1.00 | 5.00 | 3.00 | 4.00 | -0.32 | -0.65 | 0.63 | 0.33 | 0.60 | 0.963 |
| 28 | Office space allows the staff to take care of themselves when they need it. | 3.27 | 1.22 | 4.00 | 1.00 | 5.00 | 2.00 | 4.00 | -0.26 | -1.03 | 0.57 | 0.44 | 0.65 | 0.963 |
| 29 | The office includes gym equipment for physical exercise. | 1.86 | 1.22 | 1.00 | 1.00 | 5.00 | 1.00 | 2.00 | 1.37 | 0.72 | 0.22 | 0.33 | 0.50 | 0.964 |
| 30 | There is room for movement and stretching. | 3.94 | 1.03 | 4.00 | 1.00 | 5.00 | 4.00 | 5.00 | -1.13 | 0.81 | 0.74 | 0.30 | 0.54 | 0.964 |
| 31 | Office space provides an opportunity to drink tea or coffee. | 4.50 | 0.75 | 5.00 | 1.00 | 5.00 | 4.00 | 5.00 | -2.01 | 5.14 | 0.87 | 0.20 | 0.51 | 0.964 |
| 32 | A kitchen is accessible for meals. | 3.89 | 1.35 | 4.00 | 1.00 | 5.00 | 4.00 | 5.00 | -1.09 | -0.13 | 0.72 | 0.39 | 0.53 | 0.964 |
| 33 | There are areas for relaxation and sleep if needed. | 2.15 | 1.36 | 2.00 | 1.00 | 5.00 | 1.00 | 3.00 | 0.87 | -0.59 | 0.29 | 0.47 | 0.59 | 0.964 |
| 34 | Shower facilities are available. | 1.91 | 1.42 | 1.00 | 1.00 | 5.00 | 1.00 | 2.00 | 1.31 | 0.12 | 0.23 | 0.42 | 0.52 | 0.964 |
| 35 | Office spaces give people the opportunity to look out the window into the distance. | 3.81 | 1.32 | 4.00 | 1.00 | 5.00 | 3.00 | 5.00 | -0.94 | -0.42 | 0.70 | 0.36 | 0.49 | 0.964 |
| 36 | Office space allows people to grow their favorite plants if they wish. | 3.77 | 1.31 | 4.00 | 1.00 | 5.00 | 3.00 | 5.00 | -0.95 | -0.29 | 0.69 | 0.28 | 0.43 | 0.964 |
| 37 | The office space is pet-friendly. | 1.94 | 1.33 | 1.00 | 1.00 | 5.00 | 1.00 | 3.00 | 1.24 | 0.21 | 0.23 | 0.36 | 0.49 | 0.964 |
| 38 | There are quiet areas for privacy and relaxation. | 2.36 | 1.41 | 2.00 | 1.00 | 5.00 | 1.00 | 4.00 | 0.59 | -1.09 | 0.34 | 0.56 | 0.69 | 0.963 |
| 39 | Office space offers a valuable opportunity to cultivate strength and find inspiration. | 2.97 | 1.27 | 3.00 | 1.00 | 5.00 | 2.00 | 4.00 | -0.01 | -1.05 | 0.49 | 0.57 | 0.80 | 0.963 |
| 40 | Office space can accommodate guests. | 3.30 | 1.31 | 4.00 | 1.00 | 5.00 | 2.00 | 4.00 | -0.44 | -1.05 | 0.57 | 0.42 | 0.58 | 0.964 |
| 41 | The office accommodates communication with family members or children when needed. | 2.85 | 1.37 | 3.00 | 1.00 | 5.00 | 2.00 | 4.00 | 0.02 | -1.34 | 0.46 | 0.51 | 0.65 | 0.963 |
| 42 | The office space is suitable for social and cultural events. | 3.02 | 1.38 | 3.00 | 1.00 | 5.00 | 2.00 | 4.00 | -0.06 | -1.28 | 0.50 | 0.47 | 0.61 | 0.963 |
| 43 | My workplace reflects my life philosophy. | 2.90 | 1.23 | 3.00 | 1.00 | 5.00 | 2.00 | 4.00 | 0.00 | -1.03 | 0.48 | 0.49 | 0.72 | 0.963 |
| 44 | Office space represents the professional interests, achievements, and victories of employees. | 3.13 | 1.18 | 3.00 | 1.00 | 5.00 | 2.00 | 4.00 | -0.30 | -0.81 | 0.53 | 0.47 | 0.71 | 0.963 |
| 45 | Office space reflects the professional status of the employees. | 3.03 | 1.22 | 3.00 | 1.00 | 5.00 | 2.00 | 4.00 | -0.17 | -1.06 | 0.51 | 0.38 | 0.57 | 0.964 |
| 46 | Office space can store the historical records and achievements of the business. | 3.26 | 1.20 | 3.00 | 1.00 | 5.00 | 2.00 | 4.00 | -0.35 | -0.80 | 0.56 | 0.43 | 0.64 | 0.963 |
| 47 | The office provides space for pursuing hobbies. | 2.48 | 1.23 | 2.00 | 1.00 | 5.00 | 2.00 | 3.00 | 0.52 | -0.74 | 0.37 | 0.44 | 0.66 | 0.963 |
| 48 | Office space lets people listen to music if they want to. | 3.41 | 1.35 | 4.00 | 1.00 | 5.00 | 2.00 | 4.00 | -0.53 | -1.03 | 0.60 | 0.39 | 0.51 | 0.964 |
| 49 | The office gives space for reading and relaxation. | 3.00 | 1.39 | 3.00 | 1.00 | 5.00 | 2.00 | 4.00 | -0.10 | -1.37 | 0.50 | 0.46 | 0.60 | 0.963 |
| 50 | There are facilities for watching movies or listening to podcasts. | 2.70 | 1.45 | 2.00 | 1.00 | 5.00 | 1.00 | 4.00 | 0.24 | -1.39 | 0.42 | 0.47 | 0.55 | 0.964 |
| 51 | Office space reflects and reminds employees of their dreams. | 2.45 | 1.26 | 2.00 | 1.00 | 5.00 | 1.00 | 3.00 | 0.46 | -0.84 | 0.36 | 0.54 | 0.74 | 0.963 |
| 52 | I feel like my workplace is a part of me. | 3.00 | 1.27 | 3.00 | 1.00 | 5.00 | 2.00 | 4.00 | -0.16 | -1.14 | 0.50 | 0.46 | 0.66 | 0.963 |
| 53 | Those who see my workplace can learn a lot about me. | 2.84 | 1.27 | 3.00 | 1.00 | 5.00 | 2.00 | 4.00 | 0.01 | -1.18 | 0.46 | 0.36 | 0.54 | 0.964 |
| 54 | In the context of lifestyle and outlook, I find that I am akin to those who are employed in my workplace. | 3.13 | 1.13 | 3.00 | 1.00 | 5.00 | 2.00 | 4.00 | -0.47 | -0.75 | 0.53 | 0.31 | 0.48 | 0.964 |
| 55 | I love working at my workplace. | 3.61 | 1.10 | 4.00 | 1.00 | 5.00 | 3.00 | 4.00 | -0.67 | -0.28 | 0.65 | 0.42 | 0.71 | 0.963 |
| 56 | I like my workplace. | 3.51 | 1.18 | 4.00 | 1.00 | 5.00 | 3.00 | 4.00 | -0.53 | -0.69 | 0.63 | 0.47 | 0.70 | 0.963 |

*Notes*. Sk – Skew, Ku – Kurtosis, Diff – difficulty, i.e. difference of average score divided by range (maximal possible score maxscore minus minimal possible score minscore). Disc – discrimination (difference between the percent correct in the upper and lower third of the respondents tested). ITC – item-total correlation (correlation between item score and overall test score). Alpha, drop – Cronbach's alpha without a given item.
