# Supplementary material for: A new instrument to measure healthy workplace qualities: the People in the Office Scale
Source: Front Psychol. 2023 Nov 2;14:1241555. doi: 10.3389/fpsyg.2023.1241555 (PMC10658938; doi:10.3389/fpsyg.2023.1241555)
Supplement: Supplementary file 3 [file Table_3.docx]

Appendix 3

Descriptive statistics of *POS* across sex and age

|  |  | Sex | | Age | |
| --- | --- | --- | --- | --- | --- |
|  | Entire sample | Men | Women | Generation X  (41-60 years) | Generation Y  (22-40 years) |
| n (%) | 319 (100%) | 98 (30.72%) | 221 (69.28%) | 136 (53.33%) | 119 (46.67%) |
| *Workplace as a Life Narrative (WLN)* | | | | | |
| Mean (SD) | 14.82 (4.64) | 15.68 (4.72) | 14.43 (4.56) | 14.82 (4.67) | 14.54 (4.67) |
| Median [95% CI] | 15 [14-16] | 16.5 [15-17] | 14 [14-15] | 15 [14-16] | 14 [13-16] |
| Score Range | 5-25 | 5-25 | 5-25 | 5-25 | 5-25 |
| Skewness | -0.03 | -0.41 | 0.14 | -0.05 | 0.01 |
| Kurtosis | -0.62 | -0.43 | -0.53 | -0.71 | -0.58 |
| Cronbach’s alpha [95% CI] | 0.83 [0.79-0.86] | 0.85 [0.80-0.89] | 0.81 [0.76-0.85] | 0.83 [0.77-0.87] | 0.81 [0.75-0.85] |
| McDonald’s omega [95% CI] | 0.84 [0.81-0.86] | 0.85 [0.80-0.90] | 0.82 [0.77-0.85] | 0.83 [0.77-0.88] | 0.88 [0.76-0.86] |
| *Freedom of Action (FA)* | | | | | |
| Mean (SD) | 17.60 (6.81) | 18.91 (6.86) | 17.02 (6.72) | 17.59 (7.09) | 17.43 (6.59) |
| Median [95% CI] | 17 [16-18] | 19.5 [17-21] | 16 [15-17] | 17 [15-19] | 16 [15-18] |
| Score Range | 7-35 | 7-34 | 7-35 | 7-35 | 7-35 |
| Skewness | 0.40 | 0.09 | 0.55 | 0.37 | 0.47 |
| Kurtosis | -0.60 | -0.92 | -0.32 | -0.68 | -0.44 |
| Cronbach’s alpha | 0.84 [0.80-0.87] | 0.85 [0.80-0.88] | 0.85 [0.81-0.88] | 0.87 [0.83-0.91] | 0.83 [0.78-0.87] |
| McDonald’s omega | 0.84 [0.80-0.87] | 0.85 [0.81-0.88] | 0.85 [0.81-0.89] | 0.88 [0.84-0.92] | 0.83 [0.79-0.87] |
| *External Infrastructure (EI)* | | | | | |
| Mean (SD) | 15.32 (4.10) | 15.83 (3.77) | 15.09 (4.22) | 15.27 (4.17) | 15.27 (4.16) |
| Median [95% CI] | 16 [16-17] | 17 [15-18] | 16 [15-17] | 16 [15.5-17] | 16 [15-17] |
| Score Range | 4-20 | 4-20 | 4-20 | 4-20 | 4-20 |
| Skewness | -0.88 | -0.99 | -0.83 | -0.92 | -0.87 |
| Kurtosis | -0.07 | -0.60 | -0.11 | -0.12 | 0.00 |
| Cronbach’s alpha | 0.82 [0.76-0.85] | 0.78 [0.69-0.87] | 0.83 [0.77-0.87] | 0.84 [0.78-0.88] | 0.82 [0.75-0.87] |
| McDonald’s omega | 0.82 [0.77-0.85] | 0.79 [0.71-0.88] | 0.84 [0.78-0.88] | 0.84 [0.78-0.88] | 0.83 [0.76-0.88] |
| *Internal Communications (IC)* | | | | | |
| Mean (SD) | 14.72 (3.57) | 15.12 (3.46) | 14.55 (3.61) | 14.72 (3.58) | 14.50 (3.55) |
| Median [95% CI] | 15 [15-15] | 15 [15-16] | 15 [14-15] | 15 [15-16] | 15 [14-15] |
| Score Range | 5-20 | 6-20 | 5-20 | 5-20 | 5-20 |
| Skewness | -0.44 | -0.76 | -0.32 | -0.54 | -0.36 |
| Kurtosis | -0.27 | 0.32 | -0.41 | -0.09 | -0.32 |
| Cronbach’s alpha | 0.75 [0.70-0.79] | 0.74 [0.63-0.81] | 0.75 [0.67-0.80] | 0.75 [0.66-0.82] | 0.70 [0.61-0.77] |
| McDonald’s omega | 0.75 [0.70-0.79] | 0.75 [0.65-0.82] | 0.75 [0.68-0.80] | 0.75 [0.66-0.83] | 0.71 [0.63-0.78] |
| *Ergonomics (E)* | | | | | |
| Mean (SD) | 25.41 (6.29) | 26.50 (5.78) | 24.93 (6.45) | 25.57 (6.53) | 24.83 (6.19) |
| Median [95% CI] | 26 [25-27] | 28 [26-28] | 25 [24-27] | 27 [25-28] | 26 [24-27] |
| Score Range | 9-35 | 11-35 | 9-35 | 9-35 | 10-35 |
| Skewness | -0.40 | -0.82 | -0.24 | -0.46 | -0.30 |
| Kurtosis | -0.57 | 0.23 | -0.72 | -0.47 | -0.74 |
| Cronbach’s alpha | 0.88 [0.86-0.89] | 0.86 [0.81-0.90] | 0.88 [0.86-0.90] | 0.89 [0.85-0.91] | 0.86 [0.82-0.89] |
| McDonald’s omega | 0.89 [0.87-0.90] | 0.86 [0.81-0.91] | 0.89 [0.87-0.90] | 0.89 [0.85-0.91] | 0.86 [0.83-0.89] |

*Notes.* Confidence intervals for medians, Cronbach’s alphas, and McDonald’s omegas are indicated in square brackets. Confidence intervals have been estimated for each group through bootstrapping with 5,000 replicates.
