# Supplementary material for: A new instrument to measure healthy workplace qualities: the People in the Office Scale
Source: Front Psychol. 2023 Nov 2;14:1241555. doi: 10.3389/fpsyg.2023.1241555 (PMC10658938; doi:10.3389/fpsyg.2023.1241555)
Supplement: Supplementary file 4 [file Table_4.docx]

Appendix 4

Associations among the *POS* scores and the measures of attitudes towards office environment and psychological well-being

| Measures | *WLN* | *FA* | *EI* | *IC* | *E* | N | M | SD | Score range | Cronbach’s alpha |
| --- | --- | --- | --- | --- | --- | --- | --- | --- | --- | --- |
| Convergent measures | | | | | | | | | | |
| *Office Attachment Scale* | .75^***^ | .53^***^ | .29^***^ | .46^***^ | .69^***^ | 319 | 10.12 | 3.23 | 3-15 | 0.89 |
| *Perceived Restorativeness Scale TOTAL* | .66^***^ | .50^***^ | .27^***^ | .39^***^ | .52^***^ | 311 | 88.73 | 28.76 | 15-165 | 0.94 |
| Being Away | .46^***^ | .37^***^ | .12^*^ | .29^***^ | .36^***^ | 311 | 27.16 | 9.33 | 5-55 | 0.83 |
| Fascination | .58^***^ | .40^***^ | .27^***^ | .36^***^ | .46^***^ | 311 | 31.08 | 11.13 | 5-55 | 0.91 |
| Compatibility | .65^***^ | .53^***^ | .29^***^ | .36^***^ | .51^***^ | 311 | 30.49 | 12.46 | 5-55 | 0.94 |
| *Organizational Cynicism Scale TOTAL* | -.41^***^ | -.24^***^ | -.09 | -.24^***^ | -.38^***^ | 313 | 32.16 | 9.83 | 13-65 | 0.91 |
| Cognitive component | -.38^***^ | -.27^***^ | -.06 | -.30^***^ | -.39^***^ | 313 | 12.33 | 4.91 | 0-25 | 0.92 |
| Emotional component | -.39^***^ | -.23^***^ | -.10 | -.16^**^ | -.31^***^ | 313 | 7.73 | 2.88 | 0-15 | 0.90 |
| Behavioral component | -.27^***^ | -0.10 | -0.08 | -.11^*^ | -.26^***^ | 313 | 12.10 | 4.00 | 5-25 | 0.81 |
| Divergent measure | | | | | | | | | | |
| *WEMWBS* | .21^***^ | .16^**^ | .15^**^ | .16^**^ | .20^***^ | 314 | 51.37 | 7.78 | 22-70 | 0.90 |

*Notes.* WEMWBS, mental wellbeing; WLN, Workplace as a Life Narrative; FA, Freedom of Action; EI, External Infrastructure; IC, Internal Communications; E, Ergonomic. * – p<0.05; ** – p<0.01; *** – p<0.001.
